# Adaptation and Validation of the COVID-19 Stigma Instrument in Nurses:A Cross-sectional Survey

**DOI:** 10.21203/rs.3.rs-1655493/v1

**Published:** 2022-05-25

**Authors:** Feifei Huang, Wei-Ti Chen, Wenxiu Sun, Meilian Lin, Cheng-Shi Shiu, Lin Zhang, Hongzhou Lu

**Affiliations:** School of Nursing, Fujian Medical University, Fuzhou, China; University of California, Los Angeles; Shanghai Public Health Clinical Center, Fudan University, Shanghai, China; Fuqing Health School of Fujian Province, Fuqing, China; National Taiwan University, Department of Social Work, Taipei, Taiwan; Shanghai Public Health Clinical Center, Fudan University, Shanghai, China; Shanghai Public Health Clinical Center, Fudan University, Shanghai, China

**Keywords:** COVID-19, Stigma, Nurses, Psychometrics, Survey

## Abstract

**Background:**

Stigma is a prominent issue among nurses working with patients with infectious diseases, but the unavailability of validated measures of such stigma. The aim of our study was to adapt, modify, and validate the COVID-19 Stigma Instrument-Nurse -Version 3 (CSI-N-3) with both classical test theory and item response theory (IRT) analysis.

**Methods:**

We administered the scale to 249 eligible nurses who worked in a COVID-19 designed hospital in Shanghai, China.

**Results:**

The two-factor structure was confirmed by confirmatory factor analysis. The 15-item CSI-N-3 achieved Cronbach’s α of 0.64 to 0.84. Convergent validity was also demonstrated. In IRT analysis, the CSI-N-3 has ordered response thresholds, with the appropriate item difficulty and infit and outfit mean squares. Self-reported social support was the only factor influencing nurses’ COVID-19 stigma (standardized coefficients *β*=−0.21).

**Conclusions:**

The CSI-N-3 is an instrument with sound psychometric properties that can be used to measure COVID-19 stigma during the COVID-19 outbreak or afterward among nurses.

## Background

The coronavirus disease 2019 (COVID-19) global pandemic [1] has placed frontline healthcare providers (HCPs) under extraordinary stress related to the high risk of infection, understaffing, uncertainty, and psychological distress (e.g., anxiety, depression, or insomnia) related to the illness [2–3]. This is especially true for nurses, who make up the largest group of HCPs and who spend long periods providing and monitoring COVID-19 patients [4]. Nurses are often directly exposed to the virus and, therefore, are at high risk of developing the disease [4–6].

Compared with HIV, hepatitis, influenza A, H1N1, severe acute respiratory syndrome (SARS), and Middle East respiratory syndrome [7–9], COVID-19 is highly communicable and has higher mortality rates, so stigma is a prominent issue among nurses working with COVID 19 patients. Studies have shown that approximately 20–49% of nurses experienced social stigmatization in Taiwan and Singapore during the SARS outbreak [10–11], such as a nurse who was scolded by fellow passengers for making trains “dirty” [7]. In addition, Korean nurses, while caring for MERS patients, were discriminated against by family members, friends, and neighbors as well as by community members in the schools that their children attended [12]. In a similar vein, during recent COVID-19 outbreaks, nurses have suffered from COVID-19-associated stigma due to the contagious nature and the serious and potentially deadly outcomes of the disease [13]. However, there is a dearth of empirical data on how to measure the COVID-related stigma experienced by HCPs [14].

The lack of research regarding COVID-associated stigma is due to the unavailability of validated measures of such stigma. Most measures used to explore stigma of HCP during SARS [15], influenza A and H1N1 [16] were self-made assessments and did not report reliability and validity. Several existing instruments are currently being used to measure the HIV/AIDS-related stigma of people living with HIV and the general population as well as HCPs’ perceived stigma while taking care of HIV-infected individuals [17–18]. The 19-item HIV/AIDS Stigma Instrument-Nurse (HASI-N) scale was the first reliable and valid scale used to measure HIV/AIDS-related stigma that is perpetrated and experienced by nurses [18], and as the authors of that scale noted, it could potentially be adapted to address infectious diseases other than HIV/AIDS. Considering that similar stigma conditions could be experienced by HCPs who provide care to individuals with other infectious diseases, it was appropriate to modify the HASI-N to create a scale to measure COVID-19–related stigma, as we have done with the COVID-19 Stigma Instrument-Nurse (CSI-N).

Studies have shown that greater stigma is directly associated with worse mental health among HCPs caring for HIV patients in Africa [18], MERS patients in Korea [9], and SARS patients in Singapore [19], but findings regarding the linkage of stigma to HCPs’ physical health outcomes are mixed [9, 18, 20]. Furthermore, perceived stigma may impair nurses’ job satisfaction and decrease their ability to provide effective care, therefore undermining the quality of care they provided [8, 21]. However, the stigma experienced by nurses during the COVID pandemic and its influencing factors are still unknown. Limited studies have shown that COVID-19 infected people with a high level of education, perceived threats, anxiety symptoms, and familiarity with quarantined cases have a high likelihood to perceived stigma [22]. Thus, to reduce COVID-19-associated stigma and its consequences, we need a reliable and valid measure to assess the levels of COVID-19–associated stigma experienced by nurses during the pandemic as well as to understand the influencing factors of COVID-associated stigma. In this paper, we aim to explore how we: (1) adapted, modified, and validated the CSI-N with both classical test theory (CTT) and item response theory (IRT), and (2) to describe the COVID-19-associated stigma experienced by nurses and its influencing factors in Shanghai, China.

## Methods

### Participants and Setting

A sample of 249 eligible Chinese registered nurses were recruited using the convenience sampling method from 400 Chinese registered nurses working in a COVID-19–designated facility in Shanghai, China. Nurses were eligible to participate if they were rotated through COVID-related wards, understood the purpose of the survey, and were willing to participate in the survey. The sample size was determined by a subject-to-variable ratio of 5–10:1 [23], and the total number of survey variable was 11. After completing the survey, 249 participants were reimbursed for their participation and time.

### Design and Procedure

After this research was approved by the relevant institutional review boards, the study was conducted in two phases.

#### Phase I: Instrument Adaptation, Modification, and Validation

In this phase, the COnsensus-based Standards for the selection of health status Measurement INstruments checklist [24, 25] was adhered. The HASI-N [18] comprises 19 items and two factors: nurses stigmatizing patients (e.g., “*A nurse provided poorer quality care to an HIV/AIDS patient than to other patients*”) and nurses being stigmatized (e.g., “*People said nurses who provide HIV/AIDS care are HIV-positive*”). Items were rated on a 4-point Likert scale from 0 = “never” to 3 = “most of the time.” The HASI-N presented with good reliability, and the overall Cronbach’s alpha reliability was 0.90. A significant negative correlation between stigma and job satisfaction was further supported by the construct validity of the HASI-N [18].

**Figure 1:**
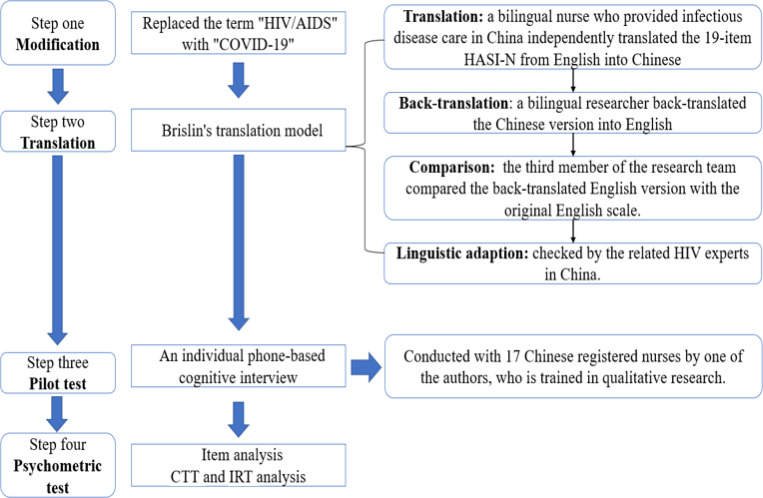
The cross-cultural adaption process the HIV/AIDS Stigma Instrument-Nurse (HASI-N)

We adapted and modified the HASI-N into the CSI-N in the four steps (see Fig. 1). In the translation step, we applied Brislin’s translation model to the cross-cultural translation [27–28]. After comparison, the Chinese phrase “*直呼护士的名字*” (“Someone will call the nurse by her first name”) in item 15 was replaced with “*耻笑护士.*” (“Someone called a nurse names”). This process yielded the Chinese version 1 of the CSI-N (CSI-N-1).

In the pilot test, an individual phone-based cognitive interview was used by structured probes to uncover how nurses interpreted items of the CSI-N-version 1 to verify its comprehensibility and readability. Example probes included: “Tell me in your own words what this question is asking,” “How did you decide on your answer to this question?” and “What does the [survey concept] mean to you?” Interviews were audio recorded and transcribed verbatim. The nurses indicated that the description of item 7 (*A nurse made a COVID-19 patient wait until last for care*) was not suitable considering the characteristics of centralized treatment and care for COVID-19 patients; therefore, we deleted that item. The 18-item Chinese version 2 of CSI-N (CSI-N-2) was then ready for validation.

After item analysis, we removed three items (I-5, I-12, and I-14) and generated the final 15 items of the CSI-N-3 (see Supplemental material A).

#### Phase II: Cross-sectional survey

In the anonymous cross-sectional survey, we adherence to the Strengthening the Reporting of Observational Studies in Epidemiology statement [26]. We used the popular Chinese online survey platform Wenjuanxing (aka, Questionnaire Star [QS], which is similar to Survey Monkey), to collect data from April 18 to May 23, 2020. We posted the recruitment information during the monthly nurses meetings at the COVID-19 facility. If nurses were interested in participating in this study and able to provide the informed consent online, we shared the QR code or the URL of the CSI-N-3 via WeChat (https://www.wjx.cn/jq/72175749.aspx, an online messaging app). Eligible nurses independently completed the 15-minute online survey, which consisted of standardized measures to assess demographics, self-reported health status, and social support, as well as the CSI-N-3. The sociodemographic variables included participants’ age, gender, marital status, ethnicity, educational levels, professional titles, and years as a nurse. The self-reported physical health, psychological health, and social support levels were measured by three questions; each of these factors was rated on a 10-point range from 1 = “very bad” to 10 = “very well.” In this study, social support was defined as nurses who received support from family, colleagues, or the hospital they worked for.

### Data Analysis

Data analyses were conducted using SPSS 23.0 (IBM, Chicago, IL, USA) and Mplus 6.1 (Muthén & Muthén, Los Angeles, CA, USA). Also, the IRT analysis was conducted using WINSTEPS 3.75.0 (Chicago, IL, USA); *p*< 0.05 was considered significant.

#### Statistical analysis of Phase I

(A). *Item analysis*: We deleted the item if it met the following criteria of CTT and IRT analysis: ①cross-loading or factor loading < 0.4, ② infit and outfit mean squares outside the range of 0.6 to 1.4; ③After items were deleted, the alpha coefficient for the overall scale was increase [29–30].

(B). *Structural validity*: We combined the confirmatory factor analysis (CFA) in CTT and IRT analysis to assess the structural validity of the scale. In the CFA, we examined the best fitting model of the scale using the method of maximum likelihood. The model’s goodness of fit was evaluated using normed χ2 (χ2/df) between 1.0 and 3.0, Root Mean Square Error of Approximation (RMSEA), Comparative Fit Index (CFI), Tucker-Lewis Index (TLI), and Normed Fit Index (NFI) [29, 31].

In IRT analysis, we examined the unidimensionality assumptions of IRT analysis by principal component analysis (PCA). Assuming nurses may interpret scales differently in terms of the items, we used the partial credit model to assess item and person separation reliability, item and person separation index, category probability curves, person-fit statistics, and test information function [30]. Infit and outfit mean squares, TIF, and differential item functioning (DIF) were assessed [32, 33].

(C). *Construct validity*: We estimated the convergent validity of the CSI-N-3 by calculating Pearson’s correlations between the CSI-N-3 and self-reported physical health, psychological health, and social support levels.

(D). *Reliability* was estimated by Cronbach’ α [31].

#### Statistical analysis of Phase II

The data met the assumptions of normality, as one-sample Kolmogorov-Smirnov tests were not statistically significant. Continuous variables were expressed as means and standard deviations (SDs), and categorical variables were expressed as proportions or percentages. We performed independent t-tests and one-way analyses of variance to identify differences in the COVID-19 stigma score among nurses. In addition, we conducted Pearson’s correlation analyses to examine the relationships among age, years of working as a nurse, and self-reported physical health, psychological health, social support levels, and the COVID-19 stigma score. Then, we conducted multiple linear stepwise regression to determine the influencing factors associated with COVID-19 stigma for nurses. Multicollinearity was assessed with the variance inflation factor.

## Results

### Sample Characteristics

A total of 249 nurses responded to the survey, and their sociodemographic characteristics are presented in Table 1.

### Psychometric Properties Of The Csi-n-3 Item retention

As shown in Table 2, according to the criteria of item retention, three items (I-5, I-12, and I-14) were removed for cross-loading (I-12, I-14), factor loading < 0.4 (I-5), and infit and outfit mean squares outside the range of 0.6 to 1.4 (I-5, I-12, I-14). After deleting I-5, the alpha coefficient for the overall scale was increased from 0.81 to 0.84.

### Structural Validity

**Figure 2:**
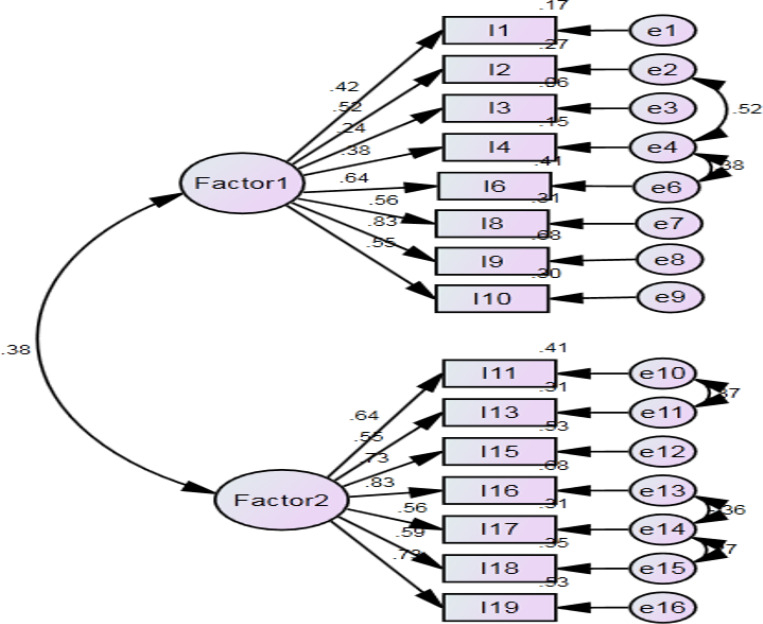
The factor structure of COVID-19 Stigma Instrument-Nurse (CSI-N-3) **Factor 1**: nurses stigmatizing patients; **Factor 2**: nurses being stigmatized.

As shown in Fig. 2, the CFA showed and confirmed that the two-factor structure model exhibited satisfactory fit to our data (χ2/df (138) = 2.12, *p* = 0.00, RMSEA = 0.06, CFI = 0.94, NFI = 0.87, & TLI = 0.93). According to the original structure of HASI-N, the two factors were labeled (a) nurses stigmatizing patients, and (b) nurses being stigmatized.

**Figure 3:**
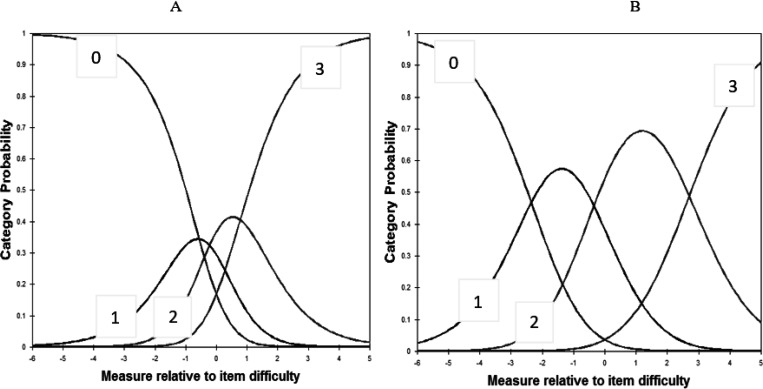
A. Category probability curves for the subscale of nurses stigmatizing patients. B. Category probability curves for the subscale of nurses being stigmatized. The four curves from left to right represent 4 response categories (0 = never; 1 = once or twice; 2 = several times; 3 = most of the time

In the IRT analysis, the two subscale’s unidimensionality assumptions were supported by PCA; that is, the residuals explained 58.9% and 55.1 % (> 50%) of the raw variance, whereas the unexplained variance in the 1st contrast was 1.7 and 2 (< 3.0) eigenvalue units. As shown in Table 3, the item difficulty for each item ranged from − 1.77 to 1.33, and infit and outfit mean squares for each item ranged from 0.32 to 1.40. No evidence of disordered thresholds was found in the category probability curves, as the category calibration increased in an orderly way (see Figs. 3A & 3B). We also found the item reliability (0.95 & 0.96), item separation index (4.15 & 5.12), person reliability (0.94 & 0.94), and the person separation index (3.92 & 3.94) in the analysis. DIF was not found when evaluated by professional title and working place[33]. Regarding the TIFs, the subscales of nurses stigmatizing patients and nurses being stigmatized gathered information most precisely when θ ranged from − 1.0 to 1.0 and − 2.0 to 2.0, respectively (see Supplemental material B).

### Convergent Validity

Pearson’s correlation analysis showed that the total CSI-N-3 score was significantly negatively correlated with self-reported physical health, psychological health, and social support levels (*r*=−0.18, −0.20, &−0.21, *p* <0.01).

### Reliability

The CSI-N-3 achieved a Cronbach’s α = 0.79 (each subscale: 0.64–0.84).

### Covid-19 Stigma Scores Of The Participants

The total mean score for the CSI-N-3 in Chinese nurses was 2.80 ± 3.73 (range 0–45) overall, with a mean score of 1.42 ± 2.13 (range 0–24) for the nurses stigmatizing patients factor and a mean score of 1.38 ± 2.46 (range 0–21) for the nurses being stigmatized factor. The mean score for each item is shown in Supplemental material C.

### Factors Associated With Covid-19 Stigma Of The Nurses

Pearson’s analysis results showed that self-reported physical health, psychological health, and social support levels were significantly correlated with the COVID-19 stigma score (*r*=−0.18, −0.20 &−0.21, *p*<0.05), whereas ages and years of working as nurses were not correlated with the COVID-19 stigma score (*r*=−0.23, & 0.01, *p*< 0.05). As shown in Table 1, other socio-demographic variables had no statistical significance (*p*< 0.05).

Thus, in the regression analysis, the COVID-19 stigma score was the dependent variable, and the statistical significance of self-reported physical health, psychological health, and social support levels were selected as independent variables (*p*< 0.05).Regression analysis showed that self-reported social support (standardized coefficients *β*=−0.206, *t*=−3.32, 95% Confidence Interval: −0.72~−0.18) was the only factor influencing nurses’ COVID-19 stigma, explaining 4.70% of the total variance (F = 5.05, *p*< 0.001). The variance inflation factor for self-reported social support was 1, which is below the criteria value of 2.1.

## Discussion

This is the first study to adapt, modify, and validate the CSI-N-3 through a rigorous, multiphase process. Psychometric evaluation based on the CTT and IRT showed that the 15-item CSI-N-3 with a 2-factor solution is a reliable and valid self-report measure for assessing COVID-19 stigma for nurses. The factor analytic strategies used in CTT shared the same factor structure model with the original scale, the HSI-N [18], including subscales of nurses stigmatizing patients and nurses being stigmatized.

In addition to the construct validity of the CSI-N, as supported by CFA, the convergent validity of the scale was also supported, as there were significant negative correlations with self-reported physical health, psychological health, and social support levels. Similar to other infectious diseases such as HIV, SARS, and MERS [9, 20], our findings showed that COVID-19 stigma adversely affects the physical and mental health of frontline nurses, although the r-value was low. The low r-value was simply shows that these constructs were significantly correlated but different in the individual constructs [34]. Besides, the Cronbach’s α was more than 0.6, indicating that the CSI-N-3 had satisfactory internal consistency and reliability [31].

Using IRT analysis, we have provided information about items in the CSI-N-3 that expand on traditional CTT methods [35–36]. Our data support that the ordered threshold in the category probability curves, which means that the category rating scale of the CSI-N-3 worked well and that nurses could use the scale to differentiate the four levels of item difficulty [30, 36]. The combination of a good person-separation index (> 2) and person reliability (> 0.8) suggests that the CSI-N-3 has acceptable measurement precision and is sensitive to distinguishing both high and low levels of social support among frontline nurses [30].

Regarding the TIF, when represented graphically, high TIF values are associated with low standard errors of measurement and can thus indicate precision [37]. The most precise information provided by the TIF for the CSI-N-3 displays the precise and reliable measure of the low to middle levels of the CSI-N-3. Furthermore, IRT measures also allow for the estimation of the equivalence of item calibrations across different samples and contexts [30]. In our study, we examined how 15 items may have been used differently, based on the nurses’ professional titles and working places. The DIF findings showed that there were no professional titles and working place differences in the item difficulty, which further support the stability and validity of the CSI-N-3[30].

The score of CSI-N-3 reflects the level of COVID-19 stigma perpetrated or experienced by nurses; however, we found that the mean score of CSI-N-3 (2.80 ± 3.73) appears to suggest a major floor effect; that is, the level of nurses stigmatizing patients or being stigmatized was not as high as the level of nurses who worked with people living with HIV (8.74 ± 9.31; [18] and MERS-CoV [9]. This finding might be explained by the cultural differences between China and South Africa. As the original study was conducted in 2008, after effective interventions to decrease stigma in healthcare institutions and nursing educations in these years, the external stigma might be decreased toward infectious diseases.

Under the influence of Confucian culture, most Chinese nurses have manifested a sense of work responsibility, dedication to patient care, personal sacrifice, and professional collegiality during the pandemic [3, 5]).

The milder forms of stigma were mainly reflected in terms of nurses being stigmatized, such as being subject to labeled as COVID-19, gossip, and as being infected and contagious. The possible explanation is that the general population, especially neighbors, routinely misunderstood nurses as a threat to the safety of others and as “disease-carriers” [38], and thus they faced avoidance by the community due to this fear [39]. Furthermore, item 3 (*A nurse who kept her distance when talking to a COVID-19 patient*) got the highest score, i.e., was most often endorsed. During the early months of the COVID-19 pandemic, personal protective equipment (PPEs) for nurses was in short supply, but nurses knew that droplet, contact, and aerosol transmissions were the main perceived infection routes of COVID-19, and thus they avoided close contact with patients when communicating with them to protect themselves. On the other hand, even with sufficient PPE, nurses showed a certain degree of fear and stigma toward COVID-19 patients.

Nevertheless, the total level of stigma was low among the nurses, and nurses were unaware that their physical distancing behaviors may have biased their provision of care [8] and exacerbated avoidance, mistreatment, and stigma toward COVID-19 patients [20].

Coinciding with similar studies [40, 41]), this study found that social support is negatively associated with COVID-19–associated stigma among nurses. This result suggests that social support is an effective coping strategy that can alleviate stigma. As Gardner and Moallef [42] suggested: support from the media and community for nurses “stalwart heroism and sacrifice” contributed to their positive experience and to less stigma [42]. As Liu et al. [3] indicated, multiple support systems, including the hospitals, colleagues, families, friends, and society, can help frontline nurses minimize the stigma associated with caring for COVID-19 patients. With logistical support from their hospital and peer support and encouragement among colleagues (e.g., the sharing of workplace experiences), frontline nurses had a sense of safety and felt less stigma [3]. But, clearly, in light of the relatively small explained variance in the regression model, further exploration of other factors that might have been included is encouraged, and including the complexity of factors that affect COVID-19 stigma for nurses is suggested.

This study has several limitations. First, this sample came from one of the premier infectious disease hospitals in Shanghai, China; therefore, it might limit the generalizability of the findings to other Chinese-speaking regions. Second, the low magnitude correlations between stigma and physical health, psychological health, and social support might be due to the three single-item physical health, psychological health, and social support measures used in this study not adequately assessing these constructs. Thus, valid and reliable scales that are available in Chinese to assess nurses’ physical health, psychological health, and social support are needed to further assess the construct validity of the scale. Third, the Chinese government is encouraging all the healthcare providers actively engaging COVID care in all the social and mass media. Since we recruited from the infectious institution in Shanghai, nurses might not have been willing to share their “true” feeling as the survey link came from their work place. A longitudinal study is recommended to see if nurses will be more forthcoming in their answers, and to compare current and future answers to see if the passage of time and the fading of the national attention to COVID-19 will affect their responses.

Furthermore, the non-significant relationship between physical and psychological health and nurses’ reported stigma may be related to measurement issues. Third, some psychometric characteristics of the CSI-N-3 could be assessed further, such as test-retest reliability, and the responsivity or sensitivity of the scale. Therefore, future longitudinal or experimental studies are warranted. Third, the sample size for IRT analysis was relatively small, despite the lack of consensus on the optimal sample size. A further refinement of the scale based on testing a larger representative sample may produce more stable parameter estimates and robust results.

## Conclusions

The preliminary psychometric properties in this paper support the use of the 15-item CSI-N-3 with two subscales as a measure of COVID-19 stigma among nurses. Although low stigma levels of nurses were found in this sample, the adverse effects of stigma during a pandemic should not be neglected. This instrument may facilitate the development of additional tailored stigma-reduction interventions. Future studies should explore how to actively mobilize nurses’ social support resources to reduce their COVID-19–associated stigma and to improve nurses’ quality of care and job satisfaction.

## Supplementary Material

Supplement 1

## Figures and Tables

**Table 1:** Sociodemographic characteristics of the nurses (*N* = 249).

Variables	N (%)	Total scores	*t/F* value	*P* value
Age (Mean ± SD)	30.79 ± 5.52			
Nursing working years	9.67 ± 6.13			
Gender				
Male	10 (4.00%)	3.30 ± 2.71	0.43	0.69
Female	239 (96.00%)	2.78 ± 3.77		
Marital status				
Single	80 (32.10%)	2.71 ± 3.53	0.16	0.93
Married	164 (65.90%)	2.85 ± 3.87		
Divorced	2 (0.80%)	1.50 ± 2.12		
Cohabited	3 (1.20%)	3.67 ± 2.31		
Educational level (Nursing Degree)				
Certificate or associate degree	142 (57.00%)	2.54 ± 3.71	1.70	0.19
Bachelor’s degree	104 (41.80%)	3.23 ± 3.76		
Master degree	3 (1.20%)	0.33 ± 0.58		
Professional title				
Newly credentialed nurses (experience less than 5 years)	108 (43.40%)	2.28 ± 2.84	1.99	0.14
Experienced nurse (experience 5–10 years)	132 (53.00%)	3.17 ± 4.33		
Charge nurse (experience more than 10 years)	9 (3.60%)	3.67 ± 3.04		
Working place				
Severe COVID cases	70 (28.10%)	2.63 ± 3.27	0.20	0.82
Mild/moderate COVID cases	66 (26.50%)	3.03 ± 3.67		
Both	113 (45.4%)	2.78 ± 4.03		
Self-reported physical health (Mean ± SD)	8.59 ± 1.85	/	/	/
Self-reported psychological health (Mean ± SD)	8.80 ± 1.73	/	/	/
Self-reported social support (Mean ± SD)	9.00 ± 1.70	/	/	/

**Table 2:** Item analysis of the scale

Item	Factor loading		Infit MNSQ	Outfit MNSQ	Cronbach’s α after removing the item^a)^	Item Retention
	Factor I	Factor II				
I-16	0.78		0.75	0.67	0.78	Yes
I-19	0.74		0.74	0.78	0.78	Yes
I-15	0.73		0.95	0.52	0.78	Yes
I-11	0.72		0.76	0.45	0.77	Yes
I-18	0.72		0.87	1.01	0.78	Yes
I-13	0.66		0.72	0.59	0.78	Yes
I-14	0.64	0.41	1.34	0.80	0.78	No
I-17	0.61		0.96	0.83	0.78	Yes
I-9		0.76	0.80	0.51	0.79	Yes
I-6		0.74	0.73	0.39	0.79	Yes
I-4		0.69	1.54	1.00	0.79	Yes
I-2		0.69	1.04	1.44	0.79	Yes
I-1		0.54	1.57	1.33	0.79	Yes
I-8		0.53	0.97	1.06	0.79	Yes
I-12	0.48	0.51	1.55	1.72	0.79	No
I-10		0.49	0.94	0.81	0.78	Yes
I-3		0.47	1.00	0.48	0.79	Yes
I-5		0.31	2.05	2.46	0.84	No

** *p* < 0.01; MNSQ: mean squares

Factor I: nurses stigmatizing patients; Factor II: nurses being stigmatized.

a Before item reduction, the overall Cronbach’s α = 0.81.

**Table 3:** The difficult, infit, outfit MNSQ and DIF of 15 items

Sub-Scales	Item	Item difficult^a^	Infit MNSQ	Outfit MNSQ	DIF contrast by professional title^b,c^		DIF contrast by working place^d,e^	
Nurses stigmatizing patients	I-1	−0.72	1.31	1.09	0.73	0.84	−0.54	−0.45
	I-2	1.07	0.93	1.40	−0.57	−0.52	−1.05	−0.28
	I-3	−1.35	0.83	1.40	−0.20	−0.11	−0.46	−0.27
	I-4	0.68	1.25	0.67	0.48	−0.11	0.27	0.96
	I-6	0.96	0.97	0.53	1.98	2.44	1.44	1.33
	I-8	−0.20	1.01	1.37	−0.76	0.02	1.78	1.36
	I-9	1.33	0.65	0.32	0.97	0.69	1.58	0.83
	I-10	0.33	1.07	1.05	−0.49	−1.02	0.86	0.17
Nurses being stigmatized	I-11	0.53	1.04	0.87	0.15	−0.30	0.19	0.00
	I-13	0.74	0.94	0.73	−0.75	0.37	−0.69	−0.29
	I-15	1.09	1.04	0.59	1.16	−0.03	−0.38	−0.64
	I-16	−0.51	1.00	0.94	0.67	1.24	0.17	0.06
	I-17	0.08	1.40	1.22	0.24	−0.69	0.31	−0.06
	I-18	−1.77	0.96	1.00	−0.33	−0.19	0.48	0.72
	I-19	−0.15	0.90	0.88	−0.64	−0.50	−0.55	−0.33

a Measured in logit; positive item logit indicates that the item requires a lower visual ability than the mean of the items and is an easier item; while a negative item logit indicates that the item requires a higher visual ability than the mean of the items and is a more difficult item; MNSQ mean square.

The DIF contrast by professional title in the following order:

b Naïve nurses compared with experienced nurse.

c Naïve nurses compared with charge nurse.

The DIF contrast by working place in the following order:

d Severe COVID cases compared with Mild/moderate COVID cases.

e Severe COVID cases compared with both.
