# Supplementary material for: Adaptation and Validation of the COVID-19 Stigma Instrument in Nurses:A Cross-sectional Survey
Source: Res Sq. 2022 May 25:rs.3.rs-1655493. Preprint. [Version 1] doi: 10.21203/rs.3.rs-1655493/v1 (PMC9164521; doi:10.21203/rs.3.rs-1655493/v1)
Supplement: Supplement 1 [file SupplementalMaterial.docx]

**Supplemental material A**

**新冠歧视工具-护士**

| 我们想要知道您作为一名护士与新冠患者一起工作的经历： | | | | |
| --- | --- | --- | --- | --- |
| 请标出在过去3个月中，您观察到的以下事情发生的频率。 | 从不 | 一次或两次 | 有几次 | 大部分时间 |
| 1.护士提供给新冠患者的照护质量较其他患者差。 |  |  |  |  |
| 2.护士对新冠患者大叫或责骂。 |  |  |  |  |
| 3.护士在与新冠患者交谈时保持距离。 |  |  |  |  |
| 4.护士无视新冠患者躯体的疼痛。 |  |  |  |  |
| 6.在科室或病房，护士不巡视新冠患者的状态。 |  |  |  |  |
| 8.为了避免接触新冠患者，护士让他自己做一些事情。 |  |  |  |  |
| 9.护士把新冠患者留在污染的床位上很长时间。 |  |  |  |  |
| 10.护士让新冠患者等待护理。 |  |  |  |  |
| 11.人们说照护新冠患者的护士是COVID-19阳性。 |  |  |  |  |
| 13.有人说照护新冠患者的护士传播了该疾病。 |  |  |  |  |
| 15.有人会因为护士照护新冠患者而耻笑护士。 |  |  |  |  |
| 16.因为为新冠患者提供照护，护士被歧视。 |  |  |  |  |
| 17.照护新冠患者的护士的配偶会害怕护士将病毒带离工作场所然后传给他／她。 |  |  |  |  |
| 18.人们说在照护新冠患者时护士会被感染。 |  |  |  |  |
| 19.人们对参与新冠照护的护士发表负面评论。 |  |  |  |  |

**Supplemental material B**

Figure 3A Figure 3B


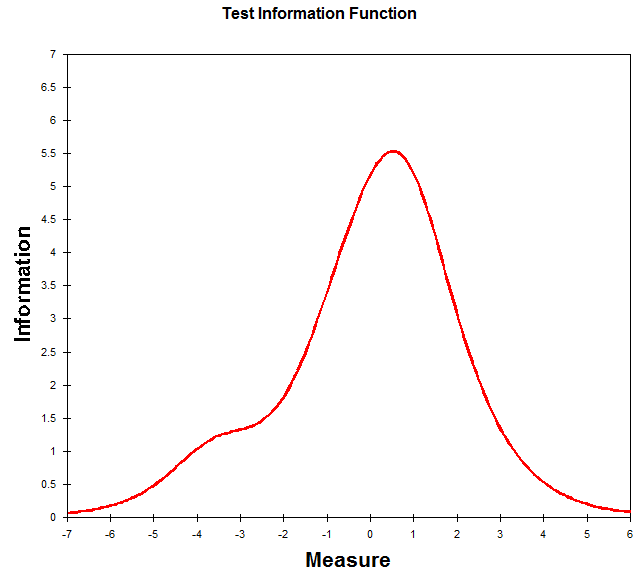

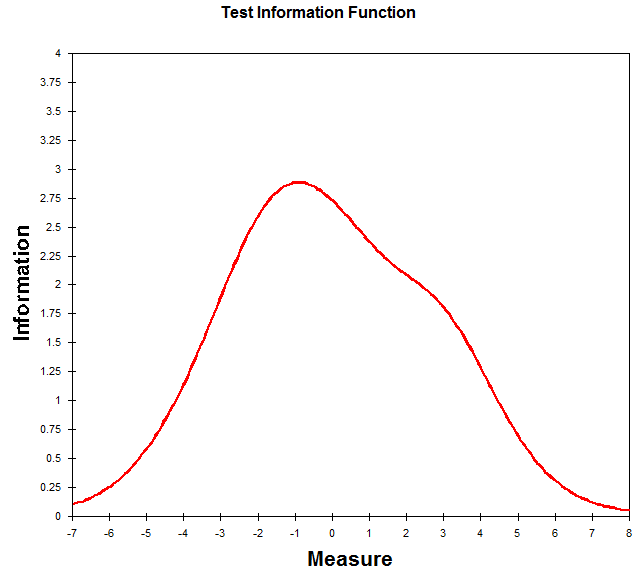


Figure 3A. Test information function for the subscale of nurses stigmatizing patients.

Figure 3B. Test information function for the subscale of nurses being stigmatized.

**Supplemental material C**

**Table 4:** Description of the final 15-item COVID-19 Stigma Instrument-Nurse

| Item | English version | Chinese version | Mean (SD) |
| --- | --- | --- | --- |
| I-3 | A nurse keep her distance when talking to a COVID-19 patient. | 护士在与新冠患者交谈时保持距离 | 0.90 (1.18) |
| I-18 | People said that nurses get infected by taking care of people with COVID-19 | 人们说在照护新冠患者时护士会被感染 | 0.41 (0.67) |
| I-16 | A nurse was stigmatized because of the COVID-19 services she  provides | 因为为新冠患者提供照护，护士被歧视 | 0.24 (0.54) |
| I-19 | People made negative remarks about nurses involved with COVID-19 care | 人们对参与新冠照护的护士发表负面评论 | 0.20 (0.46) |
| I-1 | A nurse provided poorer quality care to a COVID-19 patient than to other patients | 护士提供给新冠患者的照护质量较其他患者差 | 0.18 (0.59) |
| I-17 | The spouse of a nurse who cares for COVID-19 patients feared  that the nurse would bring the virus from work and give it to him/her | 照护新冠患者的护士的伴侣会害怕护士将病毒带离工作场所然后传给他／她 | 0.18 (0.50) |
| I-11 | People said nurses who provide COVID-19 care are COVID positive | 人们说照护新冠患者的护士是新冠阳性 | 0.14 (0.44) |
| I-8 | A nurse made a COVID-19 patient do things for himself/herself to avoid touching him/her | 为了避免接触新冠患者，护士让他自己做一些事情 | 0.12 (0.38) |
| I-13 | Someone said that nurses who care for COVID-19 patients spread the disease | 有人说照护新冠患者的护士传播了该疾病 | 0.12 (0.37) |
| I-15 | Someone called a nurse names because she takes care of COVID-19 patients | 有人会因为护士照护新冠患者而耻笑护士 | 0.10 (0.38) |
| I-10 | Nurses made COVID-19 patients wait for care | 护士让新冠患者等待护理 | 0.07 (0.30) |
| I-4 | A nurse ignored the physical pain of a COVID-19 patient | 护士无视新冠患者躯体的疼痛 | 0.05 (0.31) |
| I-6 | A nurse did not check the condition of her COVID-19 patient in the unit/ward | 在科室或病房，护士不巡视新冠患者的状态 | 0.04 (0.27) |
| I-2 | A nurse shouted at or scolded a COVID-19 patient | 护士对新冠患者大叫或责骂 | 0.04 (0.23) |
| I-9 | A nurse left a COVID-19 patient for a long time in a soiled bed | 护士把新冠患者留在污染的床位上很长时间 | 0.03 (0.19) |

SD: Standard Deviation
